# Outcomes and Cost-Benefit of a National Suicide Reattempt Prevention Program

**DOI:** 10.1001/jamanetworkopen.2025.25671

**Published:** 2025-08-06

**Authors:** Yves Gallien, Sandrine Broussouloux, Alice Demesmaeker, Anne Fouillet, Clément Mertens, Francis Chin, Guillaume Cassourret, Céline Caserio-Schonemann, Enguerrand du Roscoät, Yann Le Strat

**Affiliations:** 1 Santé Publique France, French National Public Health Agency, Saint Maurice, France; 2 Lille Neuroscience and Cognition, University Lille, Inserm, Lille, France

## Abstract

**Importance:**

Suicide attempts (SA) are a major public health concern and a preventable cause of premature death with a significant societal cost. Suicide reattempt (SR) rates are high in the postdischarge period for an SA. Brief contact interventions (BCIs) aim to prevent SR by recontacting patients after discharge through crisis cards, calls, letters, or messages. A nationwide BCI was deployed in 6 French regions between 2015 and 2017.

**Objective:**

To assess the outcomes and the cost benefit of the program in reducing SR risk within 12 months after discharge.

**Design, Setting, and Participants:**

Retrospective multicenter cohort study using nationwide data from the French health insurance database and emergency department surveillance system. Patients exposed to the program between 2015 and 2017 were matched 1:1 with unexposed patients based on age, sex, history of SA, and diagnosis codes using propensity scores and followed up for 12 months. Survival and cost-benefit analyses were conducted in [month to month] 2022.

**Exposure:**

Participation in the program, including structured follow-up using crisis cards, telephone calls, and/or postcards for up to 6 months after discharge.

**Main Outcomes and Measures:**

The primary outcome was time to first SR or suicide-related death within 12 months. The secondary outcome was the number of SRs and cost savings.

**Results:**

Among 23 146 individuals, 14 504 (62.6%) were female, 12 244 (52.9%) had no history of SA, and the mean (SD) age was 39 (17) years. Exposure to the program was associated with a lower risk of SR (adjusted hazard ratio [aHR], 0.62; 95% CI, 0.59-0.67). This association was consistent regardless of patients’ history of SAs (aHR, 0.63; 95% CI, 0.57–0.71 for those without prior attempts; aHR, 0.61; 95% CI, 0.56-0.66 for those with prior attempts) and appeared greater among female participants (aHR, 0.59; 95% CI, 0.54-0.68) than male participants (aHR, 0.68; 95% CI, 0.61-0.76). The program yielded a return on investment of €2.06 (95% CI, €1.58-€2.50) per euro spent.

**Conclusion and Relevance:**

In this cohort study, exposure to the program was associated with a reduced risk of SR and favorable economic outcomes.

## Key Points

**Question:**

Does a brief contact intervention program reduce the risk of suicide reattempts?

**Findings:**

In this cohort study of 23 146 individuals in France, the program was associated with a statistically significant 38% reduction in the risk of suicide reattempts over a 12-month period.

**Meaning:**

These findings suggest the program is a viable suicide prevention strategy, supporting its broader implementation to reduce the burden of suicide attempts in health care systems.

## Teaser

This cohort study evaluates the outcomes and cost benefit of a French national program aimed at reducing suicide reattempt risk in the 12 months after discharge.

## Introduction

Globally, approximately 700 000 deaths by suicide occur annually, alongside an estimated 16 million suicide attempts (SAs).^1,2^ Suicide is 1 of the 10 leading causes of age-standardized years of life lost worldwide.^3^ In France, despite a steady decline, suicide remains a major concern. In 2017, the crude rate was 13.4 per 100 000, higher in men and increasing with age.^4^ Moreover, there were 200 000 SA cases in France, with nearly 90 000 resulting in hospitalization.^5^ Beyond the human toll, suicide and SA also carry a high societal cost.^6,7^ By 2019, the total estimated cost of all SAs in France reached €23.9 billion.^7^ Suicide and SA are therefore a major public health concern, a preventable cause of premature death, and a burden on health care systems.

Among the risk factors for suicide and SAs, a history of SA is one of the most significant predictors of future risk.^8,9^ Individuals surviving an SA are at heightened risk for both suicide reattempt (SR) and death by suicide, particularly in the months following hospital discharge.^10,11^ These findings highlight the crucial need for targeted prevention strategies, with a particular focus on the critical postdischarge period.

Brief contact interventions (BCIs), developed in the 1980s, involve recontacting patients after hospital discharge following an SA to reduce SR risk.^12^ They are described as cost-effective, easy to implement, and capable of reaching the entire target population.^13^ BCIs complement standard care by providing support and assessing suicide risk. They can take various forms, including crisis cards with emergency contacts, helplines, phone calls, letters, postcards, or text messages.

The VigilanS program is a BCI that was first implemented in 6 regional centers in France between 2015 and 2017.^14^ Since 2023, it has been operational in all 13 regions across the country. The program provides tailored support based on prior SAs, including a resource card with an emergency contact. Individuals with a history of SAs receive phone calls over 6 months and follow-up postcards if the crisis persists or if they are unreachable by phone.

Recent literature reviews and meta-analyses of randomized clinical trials (RCTs) suggest that BCIs can reduce the risk of SR.^12,15,16^ However, various types of BCIs have been developed, combining different intervention modalities.^13^ The program represents a unique combination of interventions previously tested in the ALGOS trial.^17^ While some regional evaluations of the program have been conducted,^18^ to our knowledge, no prior study has assessed the outcomes and cost-benefit of a nationwide BCI program in clinical practice. Assessing the impact of such interventions at a national level is crucial to inform public health policies and optimize strategies for suicide prevention.

The primary objective of this study was to evaluate the association between exposure to the program and reduced SR risk, and to determine whether its potential outcomes vary based on a history of SA and sex. The secondary objective was to assess the cost-benefit of the program by estimating the cost of avoided SA for program patients in comparison with the cost of this BCI.

## Methods

This study is an independent evaluation of the program performed by the French National Public Health Agency. We conducted a national registry-based retrospective cohort study from January 1, 2015, to December 31, 2017. All the patients being followed up in the 6 program centers throughout France were considered exposed (ie, exposed to the program). We matched for unexposed patients in nationwide databases.

The program was approved by a national ethics committee. In accordance with this legal and ethical framework, patients received comprehensive oral and written information, and written informed consent was not required. This study was reviewed and approved by the Comité d’Expertise pour les Recherches, les Études et les Évaluations dans le domaine de la Santé ethics committee and the French Data Protection Authority. This study followed the Strengthening the Reporting of Observational Studies in Epidemiology (STROBE) and Reporting of Studies Conducted Using Observational Routinely-Collected Data (RECORD) reporting guidelines.^19^

### Intervention

The program is a posthospital follow-up BCI for patients who have attempted suicide. It is delivered through dedicated centers that follow a standardized protocol.^14^ After being discharged from the hospital or ED following an SA, patients are invited to join the program. Upon inclusion, each patient receives a resource card, and the referring general practitioner or psychiatrist is notified. All patients are followed up for 6 months with continued monitoring for an additional 6 months if their assessed suicide risk is high (using the Columbia Scale^20^) or in case of an SR. Patients with a history of SA (ie, at least 1 SA before the SA that led to their joining the program) are contacted between 10 and 20 days after hospital discharge. If a patient is reached by phone and appears to be in a suicidal crisis, appropriate emergency care is initiated. If patients cannot be reached by phone or if they are still in suicidal crisis during the phone call, personalized handwritten postcards are sent to them at a rate of 1 card per month for 4 months.

### Data Sources

#### Program Database

We obtained anonymized records for all program participants during the study period. Centers collected data at inclusion and follow-up, including patient characteristics, details of the index SA (date, method, history, and ED or hospitalization), and follow-up actions (calls or postcards).

#### Nationwide Databases

The French national health insurance database (Système National des Données de Santé [SNDS]) includes the national hospital and discharge database (Programme de Médicalisation des Systèmes d’Information) and the national causes-of-death registry (Centre d’Epidémiologie sur les causes médicales de Décès). Data on diagnoses and cause of death are coded using the *International Statistical Classification of Diseases and Related Health Problems, Tenth Revision (ICD-10)*.^21^ This database includes all hospital types and captures key sociodemographic (age, sex, and city of residence), care data (type of health care facility, type of care, and length of stay) and socioeconomic status (using the French deprivation index, which combines information on income, employment, education, and housing conditions based on the patient’s residential postal code).^22^ The Organisation de la surveillance coordonnée des urgences (OSCOUR) network is a nonspecific ED surveillance system.^23^ It routinely collects sociodemographic information and medical diagnoses for ED visits.

#### Data Linkage

Unlike the SNDS, the program and OSCOUR databases lack unique identifiers. We used probabilistic linkage based on SA date, facility, birthdate, residence, and discharge diagnosis. Unlinked patients were excluded. Some program cases had nonspecific *ICD-10* codes (eg, alcohol intoxication or depression), which we interpreted as underreported SAs to minimize data loss.^24^

#### Matching

To establish the population of unexposed patients, we matched program patients with patients from the national database. The matched dataset constituted our study population. See eMethods in Supplement 1 for further details.

#### Suicide Reattempt Identification

To identify SR in the 12 months after program inclusion, we searched for suicide-related deaths and suicide-related hospitalizations in the national databases (*ICD-10* codes X60-X84). SAs often involve a care pathway that includes a visit to an ED or intensive care unit followed by hospitalization. Therefore, a patient may be transferred from one ward to another on the first day of their follow-up without the transfer necessarily being due to an SR while in the hospital. To address this issue, we chose to exclude SRs occurring within the first day of the program follow-up as a suicide event.

### End Points

The primary end point was the time to the first SR or to suicide related death. Patients were censored either at 12 months of follow-up in the program or at the date of a non–suicide related death. The secondary end point was the number of SR during follow-up and the associated cost.

### Statistical Analysis

Baseline imbalances were assessed using standardized mean differences (SMD) and a propensity score model including key variables.^25^ Descriptive statistics for the study population were computed and expressed as counts with proportions and means with SDs. First, SR risk was evaluated using a Cox proportional hazards model with a frailty term for center of inclusion, adjusted for significant covariates (*P* < .20).^26,27^ The event of interest was the first SR or suicide-related death. We modeled the association with age using natural splines.^28^ Then, interaction terms were tested, and analyses were stratified to determine whether the association between the program and SR risk varied based on participants’ history of SA or their sex. Second, negative binomial regressions were performed to assess the association between variables and the number of SAs during follow-up, adjusted for significant covariates (*P* < .20). We estimated the number of SAs avoided in our study population from these model estimates. Third, cost-benefit analysis used SA 2011 cost estimates, adjusted for inflation to 2022 values to calculate the return on investment (ROI).^6^ Finally, sensitivity analyses expanded *ICD-10* codes for SR identification. In all multivariable models, statistical significance was defined as a 2-sided *P* value less than .05. See eMethods in Supplement 1 for further details. All analyses were conducted using R version 4.1.3 (R Project for Statistical Computing) in 2022.

## Results

### Participants

**Figure 1.**
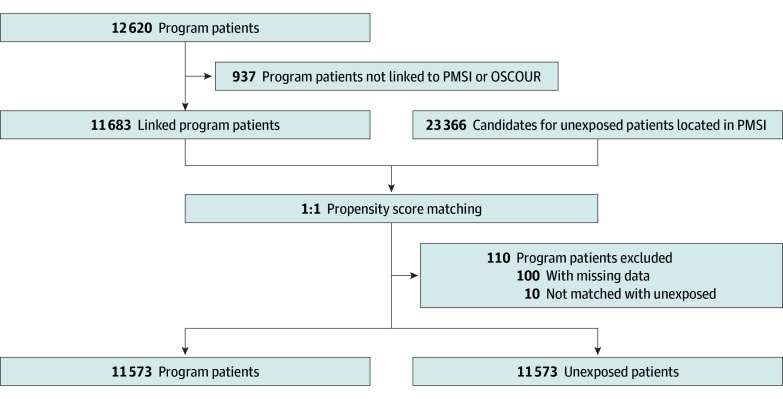
Study Flowchart OSCOUR indicates Organisation de la surveillance coordonnée des urgences; PMSI, Programme de Médicalisation des Systèmes d’Information.

From 12 620 patients included in the program, we excluded 937 patients who could not be linked and 110 who could not be matched with an unexposed patient (see Figure 1). A total of 11 573 exposed patients and 11 573 unexposed patients were included in the study (see Table 1). Of the 23 146 participants included, 14 504 (62.6%) were female, 12 244 (52.9%) had no history of SA, and the mean (SD) age was 39 (17) years. Social deprivation was quite prevalent (9476 of 23 146 patients [40.9%]).

**Table 1.** Patient Baseline Characteristics

Characteristic	Patients, No. (%)			SMD
	Program (n = 11 573)	Unexposed (n = 11 573)	Total (N = 23 146)	
Age, mean (SD), y	38.8 (16.1)	38.5 (17.4)	38.7 (16.8)	0.02
Sex				
Male	4344 (37.5)	4298 (37.1)	8642 (37.3)	<0.01
Female	7229 (62.5)	7275 (62.8)	14 504 (62.7)	
SA history				
Yes	5546 (47.9)	5356 (46.3)	10 902 (47.1)	0.03
No	6027 (52.1)	6217 (53.7)	12 244 (52.9)	
Year of inclusion				
2017	6204 (53.6)	6301 (54.4)	12 505 (54.0)	0.02
2016	3514 (30.4)	3504 (30.2)	7018 (30.3)	
2015	1855 (16.0)	1768 (15.3)	3623 (15.7)	
Social deprivation index				
1 (Least deprived)	904 (7.8)	1013 (8.8)	1917 (8.3)	0.06
2	1840 (15.9)	2069 (17.9)	3909 (16.9)	
3	1834 (15.8)	1355 (11.7)	3189 (13.8)	
4	2334 (20.2)	2321 (20.0)	4655 (20.1)	
5 (Most deprived)	4661 (40.3)	4815 (41.6)	9476 (40.9)	
Initial SA *ICD-10* code group				
Strictly related to suicide	8546 (73.8)	8284 (71.6)	16 830 (72.7)	0.05
Broader codes	3027 (26.1)	3289 (28.4)	6316 (27.3)	

Abbreviations:* ICD-10, International Statistical Classification of Diseases and Related Health Problems, Tenth Revision*; SA, suicide attempt; SMD, standardized mean difference.

During the 12-month follow-up period, we documented 4585 cases of SR or suicide-related deaths, of which 2510 occurred in the unexposed group and 2075 in the exposed group. There were 8098 SRs: 4519 in the unexposed vs 3579 in the exposed group.

### Main Results

#### Survival Analysis

**Figure 2.**
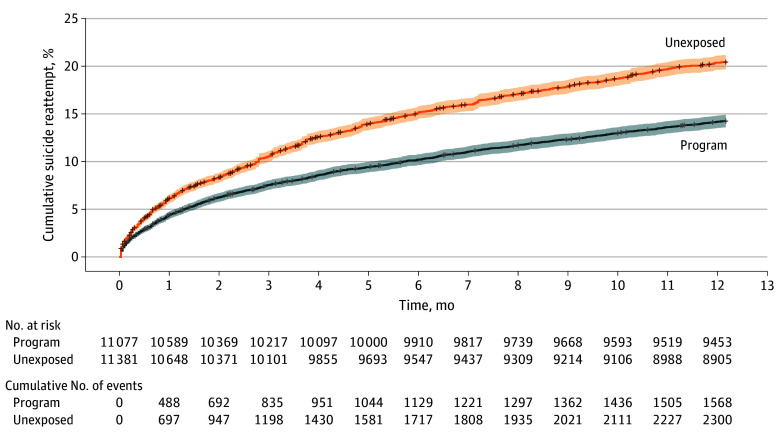
Cumulative Incidence of Suicide Reattempt During 12 Months Follow-Up by Exposure Group

Figure 2 depicts the Kaplan-Meier curve according to exposure group. After 12 months, the cumulative SR incidence was 20.4% (95% CI, 19.7%-21.2%) in the unexposed group and 14.2% (95% CI, 13.6%-14.9%) in the program-exposed groups.

In survival analyses, exposure to the program was associated with a lower risk of SR (adjusted hazard ratio [aHR], 0.62; 95% CI, 0.59-0.67) at 12 months (see Table 2). There was no sex-based difference in terms of SR risk (aHR, 0.99; 95% CI, 0.92-1.05). Patients with no history of SA were less likely to reattempt suicide (aHR, 0.40; 95% CI, 0.38-0.43).

**Table 2.** Association of Suicide Reattempt (SR) and Exposure to the Program in the 12-Month Follow-Up (N = 22 458)

Characteristic	SR, No.		SR risk, HR (95% CI)		No. of SR	No. of SR, IRR (95% CI)	
	Yes	No	Crude	Adjusted		Crude	Adjusted
Total No.	3882	18 576	NA	NA	6845	NA	NA
Exposure							
Unexposed	2307	9074	1 [Reference]	1 [Reference]	4192	1 [Reference]	1 [Reference]
Program	1575	9502	0.68 (0.63-0.72)	0.62 (0.59-0.67)	2653	0.79 (0.74-0.84)	0.76 (0.71-0.81)
Sex							
Male	1425	6955	1 [Reference]	1 [Reference]	2349	1 [Reference]	1 [Reference]
Female	2457	11 621	1.03 (0.96-1.1)	0.99 (0.92-1.05)	4496	1.11 (1.04-1.2)	1.02 (0.94-1.09)
SA history							
Yes	2469	8097	1 [Reference]	1 [Reference]	4533	1 [Reference]	1 [Reference]
No	1413	10 479	0.48 (0.45-0.51)	0.40 (0.38-0.43)	2312	0.48 (0.45-0.51)	0.41 (0.38-0.44)
Year of inclusion							
2017	2144	10 034	1 [Reference]	1 [Reference]	3839	1 [Reference]	1 [Reference]
2016	1156	5627	1.03 (0.95-1.11)	0.95 (0.88-1.02)	1948	1.06 (0.98-1.15)	1.01 (0.93-1.10)
2015	582	2915	1.01 (0.91-1.12)	0.86 (0.78-0.96)	1058	1.07 (0.96-1.19)	0.91 (0.82-1.01)
Social deprivation index							
1 (Least deprived)	384	1501	1 [Reference]	1 [Reference]	606	1 [Reference]	1 [Reference]
2	722	3099	0.94 (0.83-1.06)	0.91 (0.81-1.03)	1283	1.07 (0.93-1.24)	1.01 (0.88-1.17)
3	457	2627	0.73 (0.63-0.83)	0.74 (0.64-0.84)	855	1.00 (0.85-1.16)	0.99 (0.85-1.15)
4	793	3711	0.88 (0.78-1.00)	0.86 (0.76-0.97)	1434	1.07 (0.92-1.23)	1.00 (0.87-1.15)
5 (Most deprived)	1526	7638	0.83 (0.74-0.93)	0.81 (0.72-0.90)	2667	0.97 (0.85-1.11)	0.91 (0.80-1.04)
*ICD-10* code group							
Strictly related to suicide	3302	12 950	1 [Reference]	1 [Reference]	5957	1 [Reference]	1 [Reference]
Broader codes	580	5626	0.44 (0.40-0.48)	0.36 (0.32-0.39)	888	0.39 (0.36-0.43)	0.34 (0.31-0.37)

Abbreviations: HR, hazard ratio; *ICD-10, International Statistical Classification of Diseases and Related Health Problems, Tenth Revision*; IRR, incidence rate ratio; SA, suicide attempt.

No interaction was found between the exposure group and a history of SA, indicating that the association between program exposure and reduced SR risk was consistent regardless of patients’ history of SA. Specifically, SR risk decreased in both those without (aHR, 0.63; 95% CI, 0.57-0.71) and with prior SA (aHR, 0.61; 95% CI, 0.56-0.66). However, an interaction was observed between exposure group and sex ([data] *P* = .01). The program reduced SR risk in both male (aHR, 0.68; 95% CI, 0.61-0.76) and female participants (aHR, 0.59; 95% CI, 0.54-0.68), with a slightly greater association in female participants.

Finally, the mean (SD) number of SR over this period for persons in the unexposed and exposed groups was 0.38 (1.09) and 0.30 (1.03), respectively. Exposure to the program was associated with a lower number of SR (adjusted incidence rate ratio, 0.76; 95% CI, 0.71-0.81).

#### Cost-Benefit Analysis

We estimated the number of SR avoided in the exposed group at 1036 (95% CI, 797-1259). This represented an estimated cost saving of €5 947 485 (95% CI, €4 301 180-€6 797 960), for a reference cost of €5398, or €513 (95% CI, €372-€587) per patient included.

The total cost of the program for the 6 centers over the study period was €2 716 303, or €234 per patient for 12 months of follow-up. Therefore, the ROI calculated was €2.06 (95% CI, €1.58-€2.50) for each euro spent. Furthermore, for each patient included, being followed up in the program saved €248 (95% CI, €136-€352). eTable 1 in Supplement 1 shows the cost-benefit analysis with a reference cost updated for inflation.

#### Sensitivity Analyses

The sensitivity analysis using broader codes for identifying SR can be found in eTable 2 in Supplement 1. It showed a higher cumulative incidence of SR at 12 months (45.9% in the unexposed group vs 30.5% in the exposed group). The association between exposure to the program and reduced SR risk remained significant (aHR, 0.58; 95% CI, 0.56-0.61).

## Discussion

In this cohort study, we examined the association between inclusion in the prevention program and the risk of SR. This study represents one of the first to assess the potential outcomes of a nationwide suicide prevention program implemented in clinical practice settings. Our study has several key findings. First, exposure to the program reduced the risk of SR by 38%, after adjusting for factors associated with SR risk. Moreover, our results suggest that the program was associated with a reduced SR risk irrespective of whether the patient had a history of SA or not, and the program appeared to be slightly more effective in female patients compared with male patients. Second, the program was associated with favorable economic outcomes, with an estimated €248 in health care cost savings per patient in the year following exposure to the program.

The observed SR rate in our study population is similar to that found in the ALGOS trial.^17^ However, while the ALGOS trial did not demonstrate its effectiveness in reducing SR rates, our study revealed a significant 38% reduction in SR risk among patients exposed to the program. Differences with the ALGOS trial may be due to follow-up limitations in RCTs.^29^ In contrast, the design we used, based on exhaustive registries, addresses this issue by collecting all SR irrespective of the program center. Moreover, the clinical practice implementation of the program may have improved its effectiveness by allowing broader reach and more flexible delivery, leading to better patient engagement than in a controlled trial. Nonetheless, the absence of key clinical data, such as psychiatric diagnoses or method of SA, may also have influenced our estimates, as these variables are known to be strongly associated with SR. However, our results align with recent meta-analyses of RCTs confirming the overall potential effectiveness of BCIs in reducing SR risk.^15,16^

We observed a greater association between exposure to the program and reduced SR risk in female patients than in male patients. Similarly, previous studies on postcard interventions have similarly shown that sending postcards may be more effective in reducing SR among female patients.^30,31^ These findings may be linked to the well-documented gender paradox in suicide, which suggests that while men have higher suicide mortality rates, women tend to experience more SAs.^9,32^ This is partly due to men using more lethal methods and women attempting more often with less lethal means.^16,33,34,35^ The program, being a BCI delivered after an initial SA, may therefore be particularly effective in addressing the risk factors associated with reattempts in women.

Notably, the program reduced SR risk for both patients with and without a history of SA. Patients without a prior SA only receive a resource card, while those with a history of SA are offered additional support, including telephone calls and/or personalized postcards. The added benefit of more frequent follow-up for patients with prior SAs, through personal outreach, highlights the importance of tailored interventions that address the varying needs of individuals based on their SA history.

Finally, we demonstrated the program’s ability to reduce health care costs associated with SR. By offering potential effective, yet cost-efficient strategies, the program not only contributes to reducing the risk of SR but also alleviates the financial burden on the health care system. Further research should explore the economic impact of such programs in broader settings to confirm their cost-effectiveness across diverse health care systems.

### Strengths and Limitations

Our study has several strengths. First, the study included a large cohort from 6 program centers over 3 years, based on prior hospitalization or ED visit for SA, targeting higher-risk patients and enabling the use of electronic health records for study design. Second, this is the first evaluation of a BCI using electronic health records to assess patient outcomes. It is exhaustive, as the databases cover the entire population and national territory. Our design allowed assessment of individual-level risk differences and factors associated with SR.^36^ To avoid contamination, we excluded facilities involved in the program from the nonexposed group, preventing bias toward conservative estimates. Finally, sensitivity analyses confirmed the robustness of results under different coding assumptions.

The study also has limitations. First, the study relies partly on nationwide databases, where SAs are underreported due to nonspecific data collection.^37,38^ Nonetheless, this underreporting would have been similar for both the exposed and unexposed groups when searching for SR. We conducted a sensitivity analysis that considered broader codes; the results were similar to the primary analysis.

Second, despite propensity score matching, residual confounding cannot be ruled out. Key variables such as psychiatric diagnosis, SA method, treatment adherence, psychosocial adversity, and willingness to engage in aftercare were unavailable and may have influenced both exposure and outcome.^1,39,40^ Future studies could improve robustness by comparing program participants with national averages, or by using methods like negative control outcomes or generalized propensity scores.

Third, the program combines multiple components (crisis cards, calls, and postcards), but our data lacked details on the number and type of contacts received, preventing assessment of each component’s effectiveness. We could not account for partial adherence or incomplete delivery, nor evaluate variation in effectiveness across subgroups (eg, age or socioeconomic status). Additionally, we lacked data on individuals who declined participation, limiting assessment of selection bias. Future studies should explore which BCI components are most effective and for whom.

Additionally, nonsuicide deaths may have introduced bias. Applying competing risks models could help produce more accurate estimates. A 1-day landmark was used for immediate transfers, but no sensitivity analysis was done, which may also introduce bias.

Since 2018, the program has expanded from 6 centers to 32 across all 13 regions of France by 2023. Assessing its impact on SA incidence at the population level and reevaluating the program using more recent data—especially post-COVID-19—would be valuable.^41,42^ Finally, we used 2011 SA cost estimates, adjusted for inflation in sensitivity analyses. Further updates are needed to reflect current costs and improve the accuracy of cost-effectiveness assessments.

## Conclusions

In this cohort study of 23 146 participants, exposure to the program was associated with a reduced risk of SR and favorable economic outcomes. Integrating such programs into routine care for high-risk individuals may contribute to meaningful reductions in health care costs. Furthermore, the accessibility and low cost of the intervention make it feasible for resource-limited settings. Overall, the program highlights the importance of early, targeted interventions in suicide prevention and calls for broader adoption to enhance public health outcomes and reduce the societal impact of SAs.

## Notes

Supplement 1. **eMethods.** Additional Methodological Details
eReferences.
**eTable 1.** Program Cost Benefit Analysis **eTable 2.** Association of Suicide Reattempt and Exposure to the Program in the 12 Months Follow-Up

Supplement 2.
Data Sharing Statement

Supplement 3.
Nonauthor Collaborators
