# Supplementary material for: Outcomes and Cost-Benefit of a National Suicide Reattempt Prevention Program
Source: JAMA Netw Open. 2025 Aug 6;8(8):e2525671. doi: 10.1001/jamanetworkopen.2025.25671 (PMC12329604; doi:10.1001/jamanetworkopen.2025.25671)
Supplement: Supplement 1. — eMethods. Additional Methodological Details eReferences. eTable 1. Program Cost Benefit Analysis eTable 2. Association of Suicide Reattempt and Exposure to the Program in the 12 Months Follow-Up [file jamanetwopen-e2525671-s001.pdf]

Supplemental Online Content

Gallien Y, Broussouloux S, Demesmaeker A. Effectiveness and cost-benefit of a French national suicide reattempt prevention program. *JAMA Netw Open*. 2025;8(8):e2525671. doi:10.1001/jamanetworkopen.2025.25671

eMethods. Additional Methodological Details
eReferences.
eTable 1. Program Cost Benefit Analysis
eTable 2. Association of Suicide Reattempt and Exposure to the Program in the 12 Months Follow-Up

This supplemental material has been provided by the authors to give readers additional information about their work.

© 2025 Gallien Y et al. *JAMA Network Open*.

eMethods. Additional Methodological Details

Matching procedure: To establish the population of unexposed patients, we matched VigilanS patients with patients from the national database - drawn at random - by matching the year of inclusion in VigilanS, region of residence, sex, age group, diagnosis code used for SA hospitalization, and history of SA. We performed 1:1 nearest neighbor propensity score matching with replacement, using a caliper of 0.2 standard deviations of the logit of the propensity score, without ties.

Statistical analysis: To account for day one transfer between two hospital wards for the initial SA, we used a landmark analysis with a landmark time of one day (1). For the primary endpoint, we used a Cox proportional hazards model with time to first suicide reattempt as the event of interest. Each patient was considered to have had a reattempt only at the time of their first recurrence, which is a conservative and restrictive approach.

In both the Cox model and the negative binomial regression model, covariates with a p-value < .20 in bivariable analyses were included in the multivariable model (2,3). We modelled age using natural splines with 4 degrees of freedom, corresponding to three internal knots placed at 24, 39, and 50 years. The results of the negative binomial model are presented as an incidence rate ratio (IRR) and their 95% confidence interval, reflecting the decrease or increase in the number of SR during follow-up in the exposed group.

Finally, model assumptions were checked for both the Cox and negative binomial models. For the Cox model, the proportional hazards assumption was assessed using Schoenfeld residuals. Specifically, for the VigilanS exposure variable, the test yielded a p-value of 0.32, indicating no significant violation of the proportional hazard assumption over time. Regarding the negative binomial model, model fit was evaluated by examining the dispersion parameter and residuals. The estimated dispersion was 0.3141, indicating underdispersion.

All analyses were conducted using R (v4.1.3).

© 2025 Gallien Y et al. *JAMA Network Open*.

eReferences

1. Dafni U. Landmark analysis at the 25-year landmark point. Circ Cardiovasc Qual Outcomes [Internet]. 2011 May [cited 2025 Feb 5];4(3):363–71. Available from: https://pubmed.ncbi.nlm.nih.gov/21586725/
2. Austin PC. A tutorial on multilevel survival analysis: methods, models and applications. Int Stat Rev [Internet]. 2017 [cited 2025 Feb 5];85(2):185–203. Available from: https://pubmed.ncbi.nlm.nih.gov/29307954/
3. Babyak MA. What you see may not be what you get: a brief, nontechnical introduction to overfitting in regression-type models. 2004;

© 2025 Gallien Y et al. *JAMA Network Open*.

eTable 1. Program Cost Benefit Analysis

Panel A: Cost Benefit analysis, 2011 SR cost reference

	N. Avoided SR	Avoided Cost	ROI	Total Savings	Saving per patient
Estimate	1 035,96	5 592 128 €	2,06	2 875 825,37 €	248,52 €
Lower CI	796,81	4 301 180 €	1,58	1 584 876,88 €	136,96 €
Upper CI	1 259,35	6 797 960 €	2,50	4 081 657,18 €	352,72 €

Panel B: Cost Benefit analysis, Inflation updated SR cost reference

	N. Avoided SR	Avoided Cost	ROI	Total Savings	Saving per patient
Estimate	1 035,96	5 947 485 €	2,19	3 231 182,37 €	279,22 €
Lower CI	796,81	4 574 502 €	1,68	1 858 199,37 €	160,58 €
Upper CI	1 259,35	7 229 943 €	2,66	4 513 639,89 €	390,05 €

N: Number; SR: Suicide Reattempt; ROI: Return On Investment.

© 2025 Gallien Y et al. *JAMA Network Open*.

eTable 2. Association of Suicide Reattempt and Exposure to the Program in the 12 Months Follow-Up (n = 23 146).

Characteristic	SR[a] (Yes/No)		SR risk, HR (95% CI)[c]		N. of SR[b]	N. of SR[b], IRR (95% CI)[d]	
	Yes	No	Crude	Adjusted		Crude	Adjusted
Total number	9 832	13 314			29 066		
Exposure							
Unexposed	5 584	5 989	Reference	Reference	16 217	Reference	Reference
VigilanS	4 248	7 325	0.61 (0.58-0.63)	0.58 (0.55-0.61)	12 849	0.78 (0.75-0.82)	0.77 (0.73-0.81)
Sex							
Male	3 889	4 753	Reference	Reference	12 026	Reference	Reference
Female	5 943	8 561	0.86 (0.83-0.90)	0.88 (0.84-0.92	17 040	0.83 (0.79-0.88)	0.84 (0.80-0.88)
SA History							
Yes	5 938	4 964	Reference	Reference	20 034	Reference	Reference
No	3 894	8 350	0.46 (0.44-0.49)	0.45 (0.43-0.47)	9 032	0.40 (0.38-0.42)	0.40 (0.38-0.42)
Year of inclusion							
2017	5 386	7 119	Reference	Reference	15 977	Reference	Reference
2016	2 968	4 050	1.00 (0.95-1.05)	0.93 (0.88-0.98)	8 777	1.03 (0.97-1.10)	0.96 (0.91-1.02)
2015	1 478	2 145	0.99 (0.93-1.06)	0.86 (0.81-0.92)	4 312	1.00 (0.92-1.08)	0.91 (0.84-0.98)
Social Deprivation Index							
1 (Least deprived)	865	1 052	Reference	Reference	3 036	Reference	Reference
2	1 743	2 166	0.99 (0.90-1.08)	0.98 (0.90-1.07)	5 412	0.86 (0.78-0.95)	0.89 (0.80-0.98)
3	1 316	1 873	0.81 (0.73-0.89)	0.84 (0.76-0.92)	3 957	0.77 (0.69-0.86)	0.84 (0.76-0.93)
4	1 986	2 669	0.90 (0.82-0.98)	0.90 (0.83-0.98)	5 497	0.76 (0.68-0.84)	0.78 (0.71-0.86)
5 (Most deprived)	3 922	5 554	0.88 (0.82-0.96)	0.86 (0.80-0.93)	11 164	0.75 (0.68-0.83)	0.77 (0.70-0.84)
ICD10 Code group							
Strictly related to suicide	7 361	9 469	Reference	Reference	21 227	Reference	Reference
Broader codes	2 471	3 845	0.94 (0.89-0.98)	0.78 (0.74-0.82)	7 839	1.00 (0.95-1.06)	0.81 (0.76-0.85)

N = Number of individuals; SA= Suicide attempt

[a]SR= Suicide reattempt; [b]N. of SR = Number of suicide reattempts; [c]HR= Hazard Ratio and its 95% CI; [d]IRR= Incidence rate ratio and its 95% CI.

© 2025 Gallien Y et al. *JAMA Network Open*.
