# Supplementary material for: Outcomes and Cost-Benefit of a National Suicide Reattempt Prevention Program
Source: JAMA Netw Open. 2025 Aug 6;8(8):e2525671. doi: 10.1001/jamanetworkopen.2025.25671 (PMC12329604; doi:10.1001/jamanetworkopen.2025.25671)
Supplement: Supplement 2. — Data Sharing Statement [file jamanetwopen-e2525671-s002.pdf]

Data Sharing Statement

Gallien. Effectiveness and Cost-Benefit of a French National Suicide Reattempt Prevention Program. *JAMA Netw Open*. Published August 06, 2025.
doi:10.1001/jamanetworkopen.2025.25671

Data
Data available: No

Additional Information
Explanation for why data not available: The complete dataset of this research is only available under strictly secured conditions as it contains individual data protected by French legislation. Aggregated data could be shared upon reasonable request to Dr. Sandrine Broussouloux (sandrine.broussouloux@santepubliquefrance.fr).
