# Supplementary material for: Outcomes and Cost-Benefit of a National Suicide Reattempt Prevention Program
Source: JAMA Netw Open. 2025 Aug 6;8(8):e2525671. doi: 10.1001/jamanetworkopen.2025.25671 (PMC12329604; doi:10.1001/jamanetworkopen.2025.25671)
Supplement: Supplement 3. — Nonauthor Collaborators [file jamanetwopen-e2525671-s003.pdf]

Supplemental Online Content: Nonauthor Collaborators
*First name, last name, and suffix (if applicable) are required and will appear in PubMed.

*Group Name(s): VigilanS Collaborators							
***First Name and Middle Initial(s)**	***Last Name**	***Suffix (eg, Jr, III)**	Academic Degrees	Institution	Location (city, state/province, country)	Role or Contribution, eg, chair, principal investigator	Group (if more than 1 Group listed in the byline) and/or Subgroup (eg, Steering Committee)
Danielle	Sire		MD	**VigilanS Center of Jura**			
Claire-Marie	Tainturier		MD	**VigilanS Center of Jura**			
Michel	Walter		MD, PhD	**VigilanS Center of Brittany**			
Sofian	Berrouiguet		MD, PhD	**VigilanS Center of Brittany**			
Olivier	Guillin		MD, PhD	**VigilanS Center of Normandy (Rouen)**			
Gaël	Fouldrin		MD	**VigilanS Center of Normandy (Rouen)**			
Gérard	Boittiaux		MD	**VigilanS Center of Normandy (Pontorson)**			
Philippe	Courtet		MD, PhD	**VigilanS Center of Occitania (Montpellier)**			
Charly	Crespe		MD	**VigilanS Center of Occitania (Montpellier)**			
Guillaume	Vaiva		MD, PhD	**VigilanS Center of Hauts-de-France**			
Christophe	Debien		MD	**VigilanS Center of Hauts-de-France**			
